# A Campomelic Dysplasia A76E Mutation in Sox9 Destabilizes Protein and DNA Binding Dynamics

**DOI:** 10.3390/biom16050646

**Published:** 2026-04-27

**Authors:** Zeyaul Islam, Prasanna R. Kolatkar

**Affiliations:** Diabetes Research Center (DRC), Qatar Biomedical Research Institute (QBRI), Hamad Bin Khalifa University (HBKU), Qatar Foundation, Doha P.O. Box 34110, Qatar

**Keywords:** Sox9, HMG domain, campomelic dysplasia, pathological variant, mutational analysis, DNA binding, chondrogenesis

## Abstract

Sox9, a pivotal transcription factor belonging to the Sox family, orchestrates critical processes throughout embryonic development, maintenance and differentiation, and exerts a profound influence on organogenesis. Its regulatory versatility stems from precise binding to defined DNA regions, often in collaboration with tissue-specific partners. The dysregulation of Sox9 during chondrogenesis leads to a skeletal malformation termed campomelic dysplasia and has emerged as a significant factor in various other human diseases, including cancer. A point mutation at position 76 (alanine to glutamic acid, A76E) of Sox9 is recognized as one of the causes of campomelic dysplasia. We have used a combination of biophysical, structural and computational techniques to characterize the Sox9 A76E mutant and compare it with the wild-type (WT) Sox9. WT and A76E Sox9 assemble as homodimers, but form predominantly monomeric complexes in the presence of Sox-specific DNA. A CD analysis shows that the A76E mutant preserves the folding as well as the overall secondary structure of Sox9. Both A76E and WT Sox9 behave similarly in the presence of Sox-specific DNA. Perturbation, with increased temperature, displays a lower melting point for A76E, relative to WT Sox9, indicating decreased stability that may arise due to the long and charged side chain of glutamic acid compared to the small hydrophobic alanine, making unfavorable intra-molecular interactions. The destabilizing effect of the A76E mutant may disturb the formation of a stable higher-order complex that is a prerequisite for normal gene expression.

## 1. Introduction

Transcription factors (TFs) play an important role in gene regulation by recognizing and directly binding specific DNA regions. DNA binding initiates a cascade of events that sends additional transcriptional regulators to the promoter region and results in gene transcription [1,2,3]. TFs can also negatively regulate gene expression in various ways, such as favoring corepressors or blocking the binding of other transcription factors [4,5,6]. The Sox family of TFs plays a role in numerous developmental processes, and all Sox family members contain a characteristic DNA-binding domain known as the high-mobility group (HMG). The sex determining region Y (Sry) was the first Sox protein to be discovered, and more than 25 additional Sox proteins have since been discovered and classified based on the sequence and structural similarity of their HMG domain [7,8].

Sox9 is a member of the SRY TF family and belongs specifically to the SoxE group of the HMG-box class of DNA-binding proteins [9,10]. Sox9 is the master regulator of sex determination, and several additional critical developmental and disease-related functions of Sox9 have been identified over the past two decades [11,12,13,14]. Sox9 is essential for chondrocyte differentiation, cartilage formation, development of the pancreas, salivary glands and other organs such as the heart, brain and lung [15,16,17,18,19,20]. Therefore, genetic damage or the complete/partial loss of function of Sox9 can cause many diseases, including sexual transformation, campomelic dysplasia, pancreatic deformities, cancer and other metabolic disorders. Post-translational modification, including cofactor binding to Sox9, also plays a crucial role in modulating Sox9 functions during development [21,22,23,24]. Structurally, the Sox9 protein has an N-terminal dimerization domain, an HMG-box domain (with one export signal sequence and two nuclear localization sequences), and two transactivation domains to regulate its transcription-regulation functions [9,10]. Sox9, like other members of the HMG superfamily, regulates the transcription of many genes by binding to specific DNA target sequences via its HMG domain. The HMG domain contains approximately 70 amino acids, forms three helices and adopts a twisted L-shape that encompasses a concave surface. Binding to the target DNA region occurs through the minor groove, where helices 1 and 2 make extensive contacts with the DNA in this groove. This binding causes significant conformational changes in DNA [25,26,27]. The binding and bending of DNA allows for the recruitment of other transcription factors to nearby sites to form productive transcription complexes [28,29].

Sox9 is crucial for development and acts either as a monomer or a dimer to perform its functions depending upon specific cell types and the environment [30,31]. Bernard et al. showed that Sox9 binds as a dimer to the regulatory region of the genes involved in chondrocyte differentiation, but binds as a monomer for the genes involved in sex determination [30]. Mutations in human Sox9 cause campomelic dysplasia, a rare genetic neonatal lethal disease characterized by bone deformation and skeletal defects, such as angulation of longer bones, deformed pelvis and spine, missing ribs, craniofacial anomalies and development of male-to-female phenotypes [11,12,14,32]. Similarly, Sox9 heterozygous mice also show a similar phenotype and die after birth due to skeletal deformities [33]. Mutations in the N-terminal dimerization domain of Sox9, where the most common is A76E (alanine to glutamic acid at position 76), abolish a DNA-dependent dimer formation which, in turn, reduces the transcriptional activity of Sox9-dependent target genes [31].

Although the Sox9 point mutation A76E is known to cause campomelic dysplasia (several mutations are known to be involved in campomelic dysplasia, as summarized in Table S1), detailed insight into the associated molecular mechanism is lacking. We have initially used computational tools to analyze the effect of mutations on pathogenicity and on protein stability. We then purified the A76E and wild-type proteins for comparative studies regarding folding, secondary structure and thermal stability. We used the combined results to analyze the Sox9 interaction region with DNA and investigated the effects of A76E mutations on DNA binding. To gain insight into the structural changes, we used a modelling, intramolecular and intermolecular interaction analysis of the mutation on the stability and flexibility of Sox9 and its binding with DNA. Our results highlight the differential biochemical effects due to the A76E mutation and its implications on a disease-associated molecular mechanism. These studies lead to a better understanding of the Sox9 mutation-associated disease and future applications in potential therapeutics.

## 2. Material and Methods

### 2.1. Conservation and Predictive Analysis of A76E Using Sequence-Based Tools

Multiple sequence alignment was executed using Clustal Omega (https://www.ebi.ac.uk/jdispatcher/msa/clustalo, accessed on 1 January 2026), which incorporates seeded guide trees and HMM profile–profile techniques [34], and annotation was carried out using the Multiple alignment show web server (http://www.bioinformatics.org/sms/multi_align.html, accessed on 1 January 2026). We utilized the ConSurf server to analyze the evolutionary conservation of amino acid positions in Sox9 through empirical Bayesian inference [35]. ConSurf generates an evolutionary conservation profile by employing multiple sequence alignment and empirical Bayesian estimates, providing conservation scores on a discrete scale from 1 (most variable) to 9 (most conserved). Additionally, it categorizes amino acid residues based on solvent accessibility into exposed, buried and predicted functional residues and residues important for structural stability.

The functional impact (Deleterious/damaging) of A76E on Sox9 was predicted through several computational tools. PolyPhen-2 is a sequence-based tool that uses FASTA files as input and estimates the damaging probability of amino acid substitutions by considering comparative and physical properties. It provides a Position-Specific Independent Count (PSIC) score for mutants and calculates the deviation from wild-type [36]. A PSIC score greater than 0.09 indicates that the mutation is predicted to be deleterious. We also used EVE (evolutionary model of variant effect), a deep generative model to predict A76E pathogenicity [37]. It uses fully unsupervised deep learning trained on amino acid sequences to predict single amino acid variants of disease-related genes, and scores them in ranges from 1 (most pathogenic) to 0 (non-pathogenic). FATHMM is another tool we used for predicting the functional impact of A76E on Sox9 [38]. It features two algorithms: unweighted, which uses amino acid probabilities to identify conserved residues, and weighted, which assigns pathogenicity weights through Hidden Markov models to correlate with disease-causing amino acids. Scores near zero indicate no significant change in amino acid probabilities, where scores below zero reflect an unfavorable substitution of the mutant residue compared to the wild-type, and scores above zero represent a favorable substitution.

### 2.2. Sox9 A76E Recombinant Protein Expression and Purification

A Sox9 A76E construct was generated using standard molecular biology techniques (site-directed mutagenesis using an overlap extension PCR method). Sox9 A76E was expressed and purified as previously described for the Sox9 wild-type [24]. In short, the construct was transformed into *E. coli* BL21(DE3)-RIL chemically competent cells. An aliquot (1%) of the overnight-grown seed culture was inoculated in a fresh Luria-Bertani medium supplemented with 50 μg·mL^−1^ kanamycin and 50 μg·mL^−1^ chloramphenicol, and left to grow further until the absorbance (A) at 600 nm reached a value of around 0.8. The cultures were then incubated at 18 °C for 1 h before induction with 0.2 mM IPTG (isopropyl β-D-1-thiogalactopyranoside) for 16 h at 18 °C. The cells were collected by rapid centrifugation and resuspended in a lysis buffer (50 mM Tris-HCl, pH 8.0, 500 mM NaCl, 2 mM beta-mercaptoethanol (β-ME), and 20 mM Imadazole) along with a mixture of protease inhibitors and DnaseI. The resuspended cells were disrupted by sonication (30-s pulse on/off with 40% amplitude) (Qsonics, Newtown, CT, USA). The soluble and insoluble cell fractions were separated by centrifuging the cell lysate at 18,000 rpm for 60 min at 4 °C. The supernatant was loaded onto a nickel-nitrilotriacetic acid (Ni-NTA) column that was pre-equilibrated with an equilibration buffer (50 mM Tris-HCl, pH 8.0, 500 mM NaCl, and 20 mM Imadazole). The column was washed with 25 column volumes of wash buffer (50 mM Tris-HCl, pH 8.0, 500 mM NaCl, 2 mM β-ME, and 40 mM Imadazole) to get rid of any nonspecifically bound proteins. Finally, the protein was eluted with an elution buffer (50 mM Tris-HCl, pH 8.0, 150 mM NaCl, 2 mM β-ME, and 300 mM imidazole) and subsequently dialyzed overnight against 25 mM Tris-HCl, pH 8.0, 150 mM NaCl, 2 mM β-ME in a cold room. The dialyzed protein was further purified by gel filtration chromatography using a Superdex 200 PG 16/30 column (GE Healthcare, USA) equilibrated with 25 mM Tris-HCl, pH 8.0, 150 mM NaCl, 1 mM DTT. The concentration was measured by UV 280 nm using calculated extinction coefficients, and the purity of the protein at each stage was checked by SDS-PAGE.

### 2.3. Size Exclusion Chromatography

Size exclusion chromatography was carried out to determine the effect of the A76E mutation on the oligomeric state of the Sox9 protein using a Superdex 200 Increase 10/300 GL column, equilibrated in 25 mM Tris-HCl, pH 8.0, 150 mM NaCl, 1 mM DTT. The recombinant proteins were loaded onto the pre-equilibrated column, and chromatography was carried out at a flow rate of 0.5 mL/min. We also loaded the column with a protein–DNA complex (incubated on ice for 1 h). The two oligos, oligo 1 (5′-AACAGAACAATGGAAT-3′) and oligo 2 (5′-ATTCCATTGTTCTGTT-3′), were purchased and dissolved in water. The two DNA oligos (each is 16 base pairs) were annealed in an annealing buffer using a PCR thermocycler. The mixed oligos were heated at 95 °C for 5 min and then slowly cooled to room temperature to form a DNA duplex. We used a 1:1 molar ratio to form the protein–DNA complex. The column was calibrated with standard proteins to establish a molecular mass and elution volume relationship. The proteins used as standards were conalbumin (75 kDa), Ovalbumin (44 kDa), carbonic anhydrase (29 kDa), ribonuclease A (13.7 kDa), and aprotinin (6.5 kDa) (GE Healthcare).

### 2.4. Circular Dichroism (CD) Studies

CD spectroscopy was performed to assess the effect of the A76E mutation on the folding, secondary structure and the thermostability of Sox9 proteins. Far-UV CD spectra were collected on a Chirascan CD spectrometer (Applied Photophysics, UK) at a protein concentration of 5 μM in 25 mM Tris-HCl, pH 8.0, 150 mM NaCl, 1 mM DTT, at 22 °C using a cuvette path length of 1.0 mm and spectral collection in the range of 200–250 nm. Baselines were adjusted with a buffer, and the protein samples were scanned five times and averaged. We collected five scans of each protein, averaged and subtracted them from the buffer spectra. Thermal denaturation was monitored by measuring the change in ellipticity at 222 nm with increasing temperatures at a speed of 1 °C/min using a cuvette of path length 1.0 mm. Raw ellipticity data was converted to mean residue ellipticity before plotting. The CD spectra were also analyzed with the BeStSel (Beta Structure Selection) server [39] to estimate the content of secondary structures and native fraction in the proteins.

### 2.5. Differential Scanning Fluorimetry (DSF) Assay

For DSF studies, purified WT and A76E Sox9 proteins were used at a final concentration of 6 μM. Proteins were mixed with 3× SYPRO orange in a 0.1 mL MicroAmp Fast 96-well reaction plate (Thermo Fisher Scientific, USA). Reactions were set up in the absence or presence of Sox-specific DNA. Each sample was divided into three 50 µL replicates. Fluorescence data was collected on an Applied Biosystems Real-Time PCR system using the Quant studio 12k flex software v1.3 (Thermo Fisher Scientific). ROX (SYPRO orange) was selected as the reporter dye, and no dye was selected as a passive reference. The temperature range, to measure thermal stability, was 20 °C to 95 °C with an increase in gradient of 1 min/°C. Melting curves and differential fluorescence (−dF/dT) values were directly exported from the instrument and analyzed.

### 2.6. Modelling of Sox9 Protein and In Silico Analysis of Sox9 Structure upon A76E Mutation

Several structures of the HMG domain (DNA binding domain, common to all Sox proteins) are known in the protein data bank (PDB). The crystal structure of the HMG domain of Sox9, with its DNA sequence, is also available (PDB ID: 4S2Q and 4EUW). Our A76E mutation of interest lies outside the HMG domain, and currently, there is no structure available in PDB representing the N-terminal dimerization domain. We generated the model structure of Sox9 covering the HMG plus N-terminal dimerization domain using AlphaFold 3 [40].

To further investigate the role of the A76E mutation, we used several structure-based computational tools for a predictive in silico analysis of the impact of the mutation on the structure and function of Sox9 and its interactions with DNA. We used three computational tools for the impact of A76E, namely mCSM, SDM and CUPSAT. mCSM (mutation cut off scanning matrix) is a tool that evaluates non-synonymous mutations using a graph-based methodology to predict destabilizing mutations [41]. It employs predictive models based on atomic distance patterns of residues, correlating the impact of mutations on protein structure to atomic distances in the wild-type protein. The mCSM score (ΔΔG) suggests that mutations negatively affect protein structure if the score is below 0, thereby enhancing the understanding of disease-related mutations in various proteins. SDM (Site-Directed Mutator) assesses the stability changes between wild-type and mutant proteins through PDB coordinate files and substitution tables [42]. CUPSAT (Cologne University Protein Stability Analysis Tool) predicts protein stability shifts from point mutations by analyzing amino acid-atom potentials and torsion angles, emphasizing solvent accessibility and secondary structure [43]. We also generated the A76E mutant structure model of Sox9 for a comparative analysis of local intramolecular interactions. UCSF ChimeraX was used to visualize and perform an interaction analysis on the model Sox9 WT and A76E mutant structures [44].

## 3. Results

### 3.1. Conservation of Position 76 and Functional Effect of A76E on Sox9

We started by analyzing the conservation of position 76 to fully understand the evolutionary relations with regard to its structure and function. A multiple sequence alignment of the group SoxE family (Sox8, Sox9 and Sox10) of proteins from several organisms indicates that position 76 is well conserved and resides near the N-terminal of the HMG domain (Supplementary Figure S1a). The HMG domain is the most conserved region in Sox proteins, highlighting its critical role in DNA binding. We also analyzed the primary amino acid sequences of Sox9 from several homologs using consurf, and showed the evolutionarily conserved nature of alanine amino acid at position 76 (Supplementary Figure S1b).

We employed several computational tools to identify the structural and functional consequences of A76E mutations on Sox9. Sequence-based functional approaches were engaged to ascertain its deleterious effect. PolyPhen2 is a tool that assesses damaging mutations in amino acid sequences by categorizing mutations as possibly damaging (score 0.2 to 0.96), probably damaging (score > 0.96) or benign (score < 0.2). It predicts the potential impact of amino acid substitutions on the human protein structure and function using physical and comparative analyses. Polyphen2 predicted the substitution of alanine to glutamic acid to be damaging with a score of 1 (Table 1). We also used a deep generative model EVE (evolutionary model of variant effect) to predict mutation pathogenicity, capturing protein sequence constraints that ensure fitness through sequence variation modeling. It also predicts that the A76E mutation would be highly pathogenic. Similarly, FATHMM, a web-based tool, was used to predict the functional impact of mutations on proteins. It predicts the functional effects of protein mutations by combining sequence conservation within hidden Markov models (HMMs). Overall, all three computational tools predicted that the mutation of A76E in Sox9 is deleterious and pathogenic in nature.

### 3.2. Oligomeric Characterization of WT and A76E Sox9

**Figure 1:**
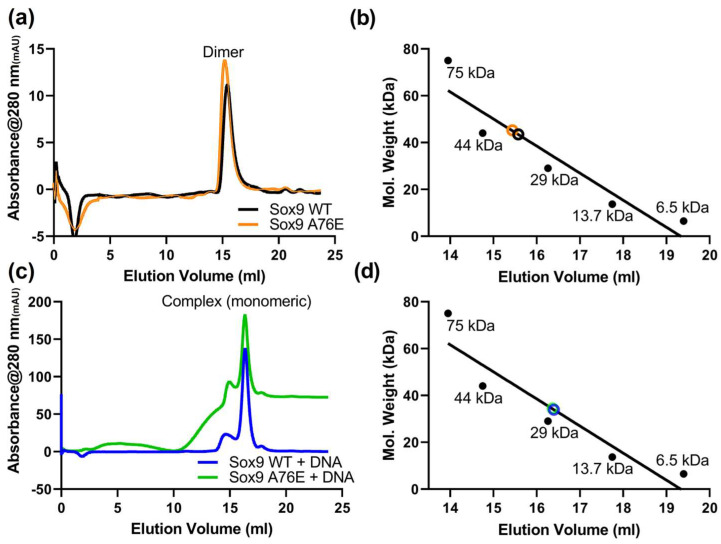
Oligomeric status of wild-type and A76E Sox9. (**a**) Size exclusion chromatography (SEC) elution profile of purified recombinant wild-type and A76E Sox9. Proteins were loaded onto Superdex 200 Increase 10/300 equilibrated with 25 mM Tris-HCl, pH 8.0, 150 mM NaCl, 1 mM DTT. (**b**) The graph shows the elution profile of standard proteins from the same column, and the calculated molecular masses of Sox9 wild-type and A76E. (**c**) SEC elution profile of wild-type and A76E Sox9 in the presence of equimolar sox-specific DNA. The protein−DNA complexes were incubated for 1 h on ice before loading on the column. (**d**) The graph shows the elution profile of standard molecular masses from the same column, and the calculated molecular masses of Sox9 wild-type and A76E in the presence of DNA.

Sox9 (WT and A76E, constructs containing HMG and N-terminal domain, 1-173 amino acids) proteins were expressed in bacteria and purified to homogeneity by a combination of affinity and gel filtration chromatography (Supplementary Figure S2). To investigate the oligomeric status, both the A76E mutant and WT Sox9 recombinant purified proteins were loaded on a Superdex 200 column and eluted. We observed a single peak in the size exclusion profile of WT and A76E Sox9 (Figure 1a). It shows the formation of homodimeric species (Figure 1b), likely due to the intact N-terminal dimerization domain in both WT and A76E mutant Sox9. The A76E mutant Sox9 showed a similar elution profile compared to WT Sox9, indicating that their homodimerization was not disturbed due to the A76E mutation.

We also analyzed the recombinant Sox9 WT and A76E proteins in the presence of equimolar Sox-specific DNA (Figure 1c). Surprisingly, we observed a peak close to around 30 kDa (Figure 1d), indicating the formation of a Sox9 (monomeric)−DNA complex. The presence of cognate DNA likely induces structural changes that dissociate the homodimer and provides a platform to bind DNA in a monomeric form. The A76E mutation, although part of the dimerization domain, may not have a role in DNA-induced monomeric−DNA complex formation.

### 3.3. Comparative Folding and Thermal Stability of A76E Sox9

**Figure 2:**
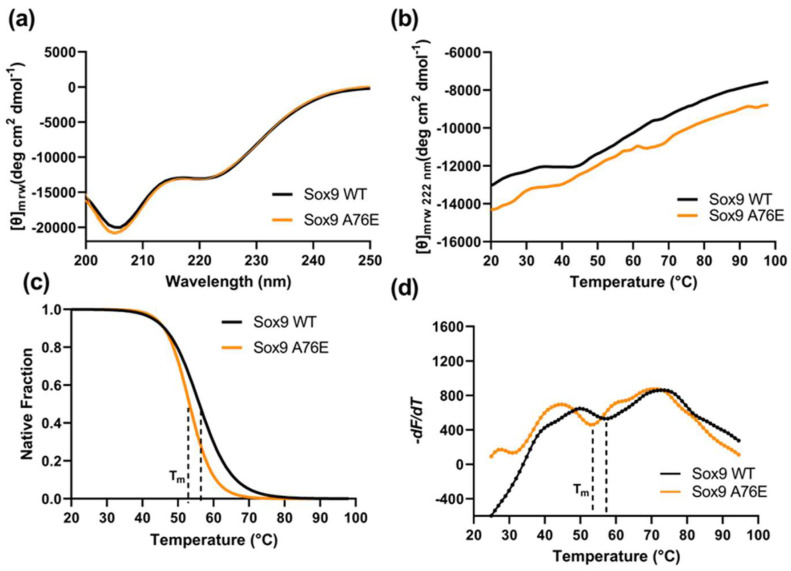
Folding, secondary structure and stability analysis of Sox9 (WT and A76E). (**a**) Far-UV CD spectra of recombinant Sox9 WT (black) and A76E mutant (orange). The proteins were 5 uM, and the CD spectra were recorded between 200 and 250 nm. Baselines were adjusted with a buffer, and the protein samples were scanned five times and averaged. (**b**) Thermal stability analysis of recombinant Sox9 WT (black) and A76E (orange), though denaturation was monitored by measuring the change in ellipticity at 222 nm with increasing temperatures at a speed of 1 °C/min. (**c**) Native fraction of the recombinant Sox9 (WT and A76E) calculated from the thermal denaturation profile as monitored at 222 nm. (**d**) Quantitative melting analysis of Sox9 WT and A76E mutant by differential scanning fluorimetry (DSF). The Sox9 proteins (6 μM) were incubated and measured in the presence of 3× SYPRO orange reporter dye. The fluorescence data were collected between 20° and 95 °C with a temperature gradient set for 1 min/°. The first derivative of the progress curves, differential fluorescence (−dF/dT), provides a means to determine Tm values. The measurements were performed in triplicate.

To investigate the significance of the A76E substitution on the Sox9 structure, the secondary structure of the A76E mutant was compared with the Sox9 WT. Circular dichroism (CD) spectroscopy was employed to assess the secondary structure, as well as the folding status of both Sox9 proteins. The results of CD measurements indicated that the Sox9 WT and Sox9 A76E were well folded with similar secondary structure features (Figure 2a). The calculated percentage of the secondary structure content also agreed well with 28–29% in helices (Supplementary Figure S3). We also compared WT/A76E Sox9 with Sox9 HMG to ascertain the helical content of the N-terminal dimerization domain. Compared to HMG, we observe a sharp decrease in helical content (HMG domain shows 61.5% helical content) with a substantial increase in the unstructured random coil region (∼55% unstructured region in WT/A76E Sox9, ∼37% unstructured region in Sox9 HMG), suggesting that the N-terminal dimerization domain has a significant intrinsically disordered/unstructured region (Supplementary Figure S3).

We further probed the thermal stability of the Sox9 A76E compared to the WT using CD with increasing temperatures (Figure 2b). A structural stability change can be monitored by gradually increasing the temperature, which causes protein unfolding and the loss of the tertiary/quaternary structure. The thermal stability, as indicated by the CD spectra at a 222nm wavelength and increasing temperatures, shows that the Tm of the A76E mutant was ∼4 °C lower than the WT Sox9 (Figure 2b,c). The A76E mutant was found to be more susceptible to thermal denaturation than the WT Sox9, indicating its relative structural instability. We also implemented differential scanning fluorimetry (DSF) to assess the A76E effect on Sox9 stability. DSF utilizes SYPRO orange dye (probes hydrophobic patches as proteins unfold), where the Sox9Wt or Sox9 A76E samples were gradually heated from 20° to 95 °C to calculate their melting points. The Sox9 A76E mutant protein had a Tm of 53.7 °C, while the Tm for the Sox9 WT shifted to 57.2 °C, indicating a decrease in stability for the mutant protein (Figure 2d).

**Figure 3:**
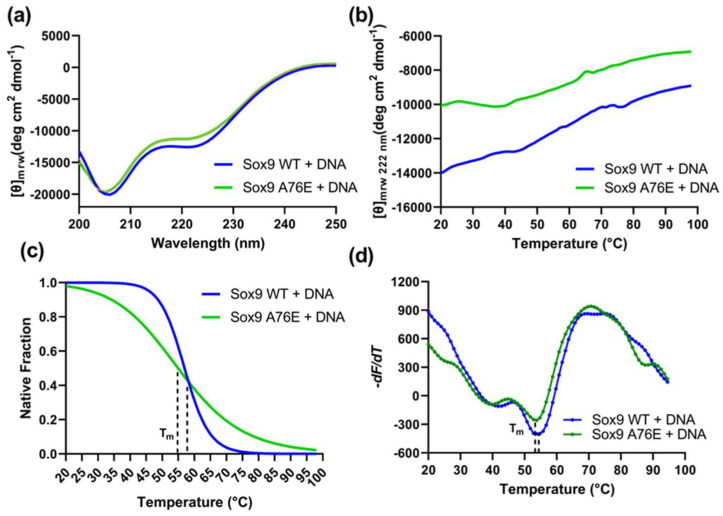
Secondary structure and stability analysis of Sox9 (WT and A76E) in the presence of sox-specific DNA. (**a**) Far-UV CD spectra of recombinant Sox9 WT (blue) and A76E mutant (green) in the presence of sox-specific DNA. The equimolar amount of protein and DNA was mixed, and the CD spectra were recorded between 200 and 250 nm. Baselines were adjusted with a buffer, and the protein samples were scanned five times and averaged. The DNA spectra were further subtracted to get only the protein contribution. (**b**) The thermal denaturation profile of recombinant WT (blue) and A76E Sox9 in the presence of sox-specific DNA. CD at 222 nm was measured with increasing temperatures. (**c**) The fraction of folded proteins was calculated by measuring the change in ellipticity at 222 nm with increasing temperatures. (**d**) DSF melting profile of recombinant Sox9 WT and A76E mutant in the presence of sox-specific DNA. Sox9 proteins were incubated in the presence of DNA, 3× SYPRO orange dye was added, and the fluorescence data were collected in triplicate between 20° and 95 °C with the temperature gradient set for 1 min/°C.

A secondary structure analysis of the Sox9 WT and A76E in the presence of DNA was also performed by CD. Far-UV CD spectra indicated that DNA did not change the secondary structure profile (Figure 3a), and it followed a similar pattern as observed in the absence of DNA (Supplementary Figure S2). Thermal denaturation using CD and fluorescence-based DSF revealed that the presence of DNA has no effect on the stability of the WT or A76E Sox9 proteins (Figure 3b–d). The WT Sox9 was structurally more stable than the A76E mutation, both in the absence and presence of DNA. These results suggest that the substitution of A76, with a large and charged residue, can destabilize the Sox9 protein without affecting its DNA-binding capability.

### 3.4. Sox9 Modelling and Predicting the Impact of A76E on Sox9 Stability

**Figure 4:**
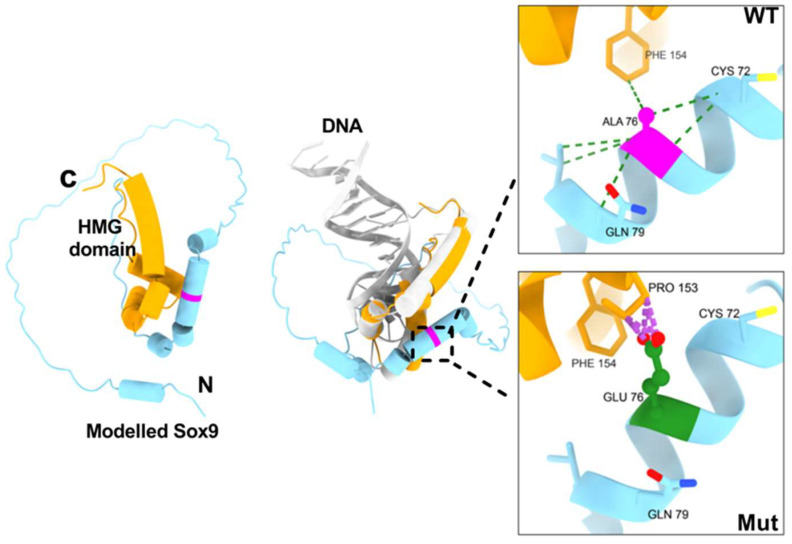
Overall structure of the modelled Sox9 and a mutational analysis of A76E. Cartoon representation of Sox9 HMG and dimerization domain (**left** panel). The HMG domain is colored orange. Structural superimposition of the modelled Sox9 with Sox9 HMG−DNA complex structure (PDB ID: 4S2Q), highlighting the position of the A76E mutation (**middle** panel). The WT residue is shown as magenta, while the mutated residue is green. Interactions are shown as a dotted line (**right** panel).

We have generated the model of full-length Sox9 protein using AlphaFold 3 (we predominantly focus on the structure model of amino acids 1-173, covering the N-terminal dimerization and HMG domain). The modelled structure of Sox9 shows a well-defined HMG domain, known to bind DNA and is a central feature of all known Sox proteins. The HMG domain has three alpha-helices adopting an L-like topology (Figure 4). The N-terminal region harboring the dimerization mutation mainly shows an unstructured region, although the A76E mutation is located in the helix adjacent to the HMG helices (Figure 4). A comparison of our modelled Sox9 structure with the Sox9 HMG−DNA complex structure (PDB ID: 4S2Q) reveals that HMG adopts a similar topology and helix, whereas the A76E mutation may not directly be in contact with DNA (Figure 4). Our SEC results show that Sox9 exists as a dimer in the absence of DNA using the N-terminal dimerization domain. DNA binding through the HMG domain likely alters the adjacent dimerization domain. Overall, DNA binding appears to influence the dimerization, allowing predominantly the Sox9 (monomer)−DNA complex.

Our SEC studies indicated that Sox9 exists as a homodimer in the solution, and to investigate further, we modelled the Sox9 dimer using AlphaFold 3. Each monomer shows an extended conformation where the HMG domain of one monomer interacts with the dimerization domain of the second monomer (Supplementary Figure S4a). A structural comparison of our modelled Sox9 dimeric structure, with the Sox9 HMG−DNA complex structure (PDB ID: 4S2Q), reveals similar structural features of the HMG domain (Supplementary Figure S4b). As the Sox9 HMG−DNA complex structure lacks the N-terminal dimerization domain, we also modelled the Sox9−DNA complex to observe the N-terminal dimerization domain in the presence of DNA (Supplementary Figure S4c). It shows that the dimerization interface observed in the Sox9 dimer was not conserved and extended from the HMG domain. A comparison of the modelled Sox9 dimer with the Sox9−DNA complex highlighted that DNA may hinder the dimer interface (Supplementary Figure S4d).

We have used several computational structure-based predictors to calculate the difference in free energy of the A76E mutation: delta delta G (ΔΔG). The mutation at position 76 (alanine to glutamic acid; A76E) results in a ΔΔG of −2.0 to −3.0 kcal/mol, highlighting that the mutation severely reduces protein stability (Table 1).

### 3.5. Structural Analysis of A76E Mutant on Sox9 Interactions

The effect of intramolecular interactions of the A76E mutation was analyzed by computing H-bonding, contacts and clashes in the wild and A76E Sox9 structure. UCSF ChimeraX (v 1.2.5) was used to generate the A76E models of Sox9 for the corresponding amino acid substitutions.

The helix of the N-terminal dimerization domain of Sox9 harbors the A76E mutation. A comparative interaction analysis of the mutant structure with wild-type reveals that the longer side chain of glutamic acid results in several clashes within the vicinity of A76E, noticeably with proline 153. In contrast, WT alanine makes a favorable hydrophobic interaction with phenylalanine 154 of the HMG domain (Figure 4). In the wild-type, alanine 76 makes two hydrogen bond-based interactions with valine 80 and cysteine 72. The two WT hydrogen bonds are made through main-chain interactions, and thus, the mutation effects could be installed by a change in local structure, which affects main-chain binding. In the future, crystal structures of Sox9 WT and A76E mutant full-length or HMG + N-terminal dimerization domain in the absence and presence of DNA are needed to make a more accurate conclusion. Overall, the mutant A76E Sox9 protein shows less stability compared to the wild-type Sox9 protein.

## 4. Discussion

Sox9, like other members of the Sox family, is crucial in gene regulation, affecting the expression of genes related to developmental processes, sex determination and organ development. Sox9 functions as either a monomer or a dimer, with its activity influenced by post-translational modifications. The majority of the functional complexity derives from the ability of its HMG domain to bind DNA in various combinatorial modes. Sox proteins contain other domains and regions apart from the HMG domain that give them a group-specific identity and functional repertoires. Mutations in Sox9 can lead to disorders like campomelic dysplasia, especially in its N-terminal dimerization domain. In this study, we characterized the A76E mutation associated with campomelic dysplasia and located it in the N-terminal dimerization domain near the HMG domain.

Sox9 protein binds target genes as a monomer or dimer to perform its transactivation functions depending upon specific cell types and the environment [30,31,45]. It has been demonstrated that Sox9 proteins homodimerize upon binding to inverted recognition sites present in the enhancers or promoters of their target genes. Sox9 binds cooperatively as a dimer to DNA enhancer elements in genes related to chondrocyte differentiation, such as Col11a2 and Col9a2. However, it binds as a monomer to the regulatory region of the sex-determining gene SF1 [30]. Dimerization and the ability to activate promoters are compromised in mutant SOX9 proteins, while other functional features remain unchanged [30,31]. A76E mutation does not affect DNA binding and the activation of the SF1 enhancer [30]. Our results, in contrast to previous studies, reveal that neither dimerization nor DNA binding was affected due to the A76E mutation. DNA binding to the Sox9 HMG domain induces disruption of the self-assembled homodimeric state. Sox9 exists as a homodimer due to the presence of the N-terminal dimerization domain, but it favors formation of the monomeric−DNA complex, prompted by DNA binding. The A76E mutation may not have a role in dimerization or formation of monomeric−DNA species in the presence of DNA binding, but it creates a more general phenomenon where it may affect the recruitment of other transcription factors upon monomeric−DNA binding or additional proteins required for the expression of downstream genes.

The secondary structure of the Sox9 WT and A76E proteins was determined by CD to monitor conformational changes caused by the mutation. Based on CD spectra, A76E mutations shared a similar CD pattern to that of the WT protein, showing the characteristic of α-helical proteins containing an unstructured random coil at the N-terminal. This indicates that the A76E mutation has no significant effect on the secondary structure of the protein. We also analyzed the conformational change at the level of the secondary structure of the protein in the presence of DNA, and found no change in the overall structure with the addition of DNA. It enforces the earlier studies showing that A76E does not affect DNA binding. However, thermal stability studies using CD and DSF show a marked decrease in the melting point of the Sox9 A96E mutant. Overall, the stability analysis indicated that the A76E mutation caused protein structural destabilization, likely contributing to unstable protein−DNA complexes, which require additional cofactors. Sox9 homodimer recruits Sox5 and Sox6, and the complex activates the Col2a1 and other chondrogenic genes that are required for chondrogenesis [46,47]. Contrarily, Sox9 facilitates the binding of Gli2 and Gli3 at the nearby conserved Gli-binding element to repress the gene expression of Col10a1, which is a marker for chondrocyte maturation in hypertrophic chondrocytes [48]. Sox-trio complex also directly interacts with Runx1 (Runt domain transcription factor 1) to enhance the activation of chondrocyte/cartilage, the maintenance gene required for the cartilage matrix production [49]. Sox9 works at several levels for the recruitment of other proteins to form stable transcriptional complexes, and the A76E mutant destabilization may affect the recruitment for stable higher-oligomeric complexes.

HMGs (present in all Sox proteins), the main DNA-binding domain, have been studied extensively. Several structures have already been determined from a number of Sox family members, including Sox9. Sox proteins are believed to achieve functional specificity either through structural rearrangement of the HMG-domain arms or by inducing specific kinks in the DNA [25,26,27]. The specificity of Sox proteins might also be achieved via bending DNA to distinctive degrees, which might subsequently recruit Sox protein-specific cofactors for partnering, and establish higher-order transcriptional complexes [28,29]. The crystal structure of the HMG domain of Sox9, with its DNA sequence, is available (PDB ID: 4S2Q and 4EUW). A structural analysis of the available Sox9 HMG-domain structures (complexed with cognate DNA) showed that both mouse (4S2Q) and human (4EUW) Sox9 structures are highly similar. As our A76E mutation is located outside the HMG domain, we modelled the Sox9 structure to evaluate the effect of this mutation on its structure. A structural analysis highlighted the proximity of the A76E position to the HMG helix that binds to DNA. Our in silico analysis also predicted that the A76E mutation severely destabilizes the Sox9 structure, possibly through disruption of well-established hydrophobic interactions and clashes with neighboring amino acid residues.

The structure modelling of the Sox9 dimer highlighted that the HMG domain of one monomer interacts with the N-terminal dimerization of the neighboring monomer, and the presence of DNA may disrupt the dimerization ability of Sox9.

## 5. Conclusions

Collectively, our results emphasize that the WT and A76E mutant Sox9 both exist as homodimers, possibly through the helix region in the N-terminal dimerization domain. Sox9 A76E shows a similar propensity to bind DNA as compared to wild-type Sox9 in terms of forming complexes with DNA. The destabilizing effect of the A76E mutant on Sox9 structure arises due to long and charged side chains of glutamic acid that likely disrupt the alanine-based hydrophobic intramolecular interaction network, as well as possible steric clashes. Transitions of SoxA76E upon condition changes could potentially affect the functions of Sox9, playing a significant role in a higher-order Sox9−DNA complex involving other cofactor proteins. The trickle-down effect of local destabilization may affect the formation of stable higher-order complexes at the promoter or enhancer regions, which are required for proper gene expression of downstream genes.

## Figures and Tables

**Table 1:** Predicted Sox9 structure, function and protein stability scores of the A76E mutant by the three sequence-based and three structure-based predictors. ΔΔG values of energies are given in kcal/mol. ΔΔG < 0 indicates a reduction in protein stability [41,42,43].

	Mutation	Predicted Score			Outcome/Overall Stability
Sequence-based predictors		PolyPhen -2	EVE	FATHMM	
	WT	00	00	00	-
	A76E	1.000	0.995	−7.99	Deleterious/pathogenic
		Predicted ΔΔG (kcal/mol)			
Structure-based predictors		mCSM	SDM	CUPSAT	
	WT	00	00	00	-
	A76E	−2.00	−3.06	−3.10	Destabilizing

## Data Availability

The original contributions presented in this study are included in the article/Supplementary Material. Further inquiries can be directed to the corresponding author.

## Supplementary Materials

The following supporting information can be downloaded at: https://www.mdpi.com/article/10.3390/biom16050646/s1, Figure S1: Multiple sequence alignment and conservation of Sox9 sequence; Figure S2: Purification of Sox9 WT and A76E proteins; Figure S3: Folding and secondary structure contents of Sox9 WT, Sox9 A76E and Sox9 HMG; Figure S4: Structure models of Sox9 dimer and Sox9-DNA complex; Table S1: Sox9 mutations involved in Campomelic dysplasia.
