# Supplementary material for: A Campomelic Dysplasia A76E Mutation in Sox9 Destabilizes Protein and DNA Binding Dynamics
Source: Biomolecules. 2026 Apr 27;16(5):646. doi: 10.3390/biom16050646 (PMC13204968; doi:10.3390/biom16050646)
Supplement: Supplementary file 1 [file biomolecules-16-00646-s001.zip › biomolecules-4229229-supplementary.pdf]

SUPPLEMENTARY INFORMATION

A Campomelic Dysplasia A76E Mutation in Sox9 Destabilizes Protein and DNA Binding Dynamics

Zeyaul Islam, Prasanna R. Kolatkar *

Diabetes Research Center (DRC), Qatar Biomedical Research Institute (QBRI), Hamad Bin Khalifa University (HBKU), Qatar Foundation, Doha P.O. Box 34110, Qatar
* Correspondence: pkolatkar@hbku.edu.qa

Table S1

Figure S1

Figure S2

Figure S3

Figure S4

Table S1: Sox9 mutations involved in Campomelic dysplasia.

Amino acid position	Amino acid change	Impact of mutation
76	A>E	Dimerization and the resulting capacity to activate promoters
108	P>L	Loss of DNA binding
112	F>L	
112	F>S	
113	M>V	Residual DNA binding and transactivation of regulated genes
119	A>V	Almost no loss of DNA binding
143	W>R	
152	R>P	
154	F>L	Reduction in DNA binding activity
158	A>T	Reduction in nuclear import efficiency
165	H>Q	Residual DNA binding and transactivation of regulated genes
165	H>Y	Loss of DNA binding
169	H>P	Decreased transactivational activity
170	P>L	
173	K>E	

Figure S1.

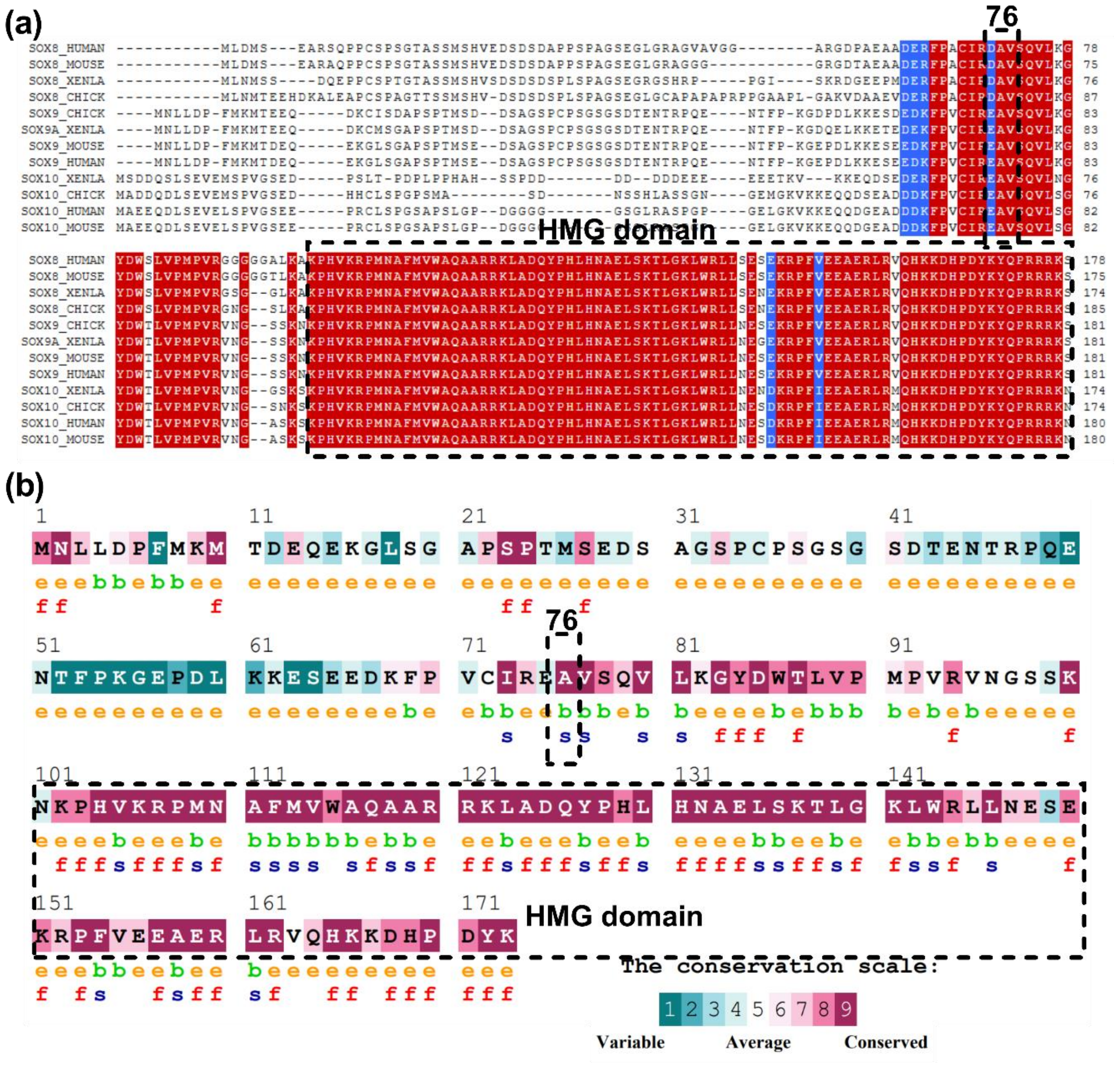

FIGURE S1. Multiple sequence alignment and conservation of Sox9 sequence. (a) Sequences of Sox9 from several homologs were aligned (*Homo sapiens, Mus musculus, Gallus gallus, and Xenopus laevis*). SoXE group of proteins including Sox8 and Sox10 were also aligned. Multiple sequence alignment of these proteins highlighted the high sequence conservation of position 76. HMG domain was shown as the most conserved region in these proteins. Identical amino acids are shown in red while similar amino acid residues are shown in blue. (b) Evolutionary conservation of Sox9 amino acid sequence as analysed by Consurf. The color-coding scheme indicates the conservation profile of amino acids, e- an exposed residue, b- a buried residue, f- a predicted functional residue (highly conserved and exposed), s- a predicted structural residue (highly conserved and buried).

Figure S2.

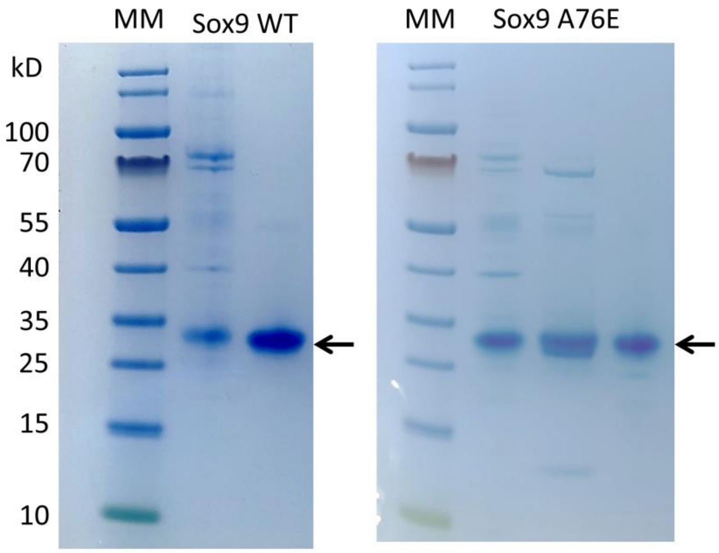

Figure S2: Purification of Sox9 WT and A76E proteins. SDS-PAGE of purified Sox9 (containing HMG and N-terminal dimerization domain) WT and A76E mutant showing the purity of the proteins.

Figure S3.

(a)

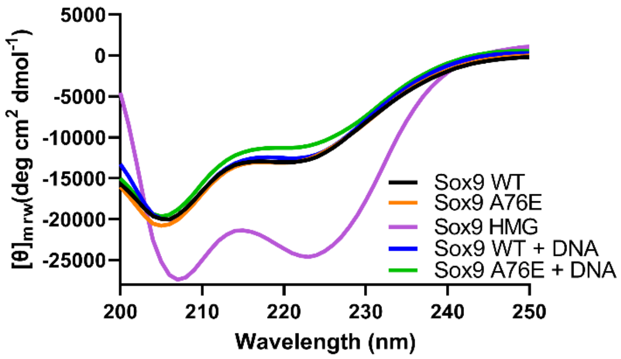

(b)

	Structured		Unstructured	
**Proteins**	**Helix**	**B-sheet**	**Turn**	**Others**
Sox9 WT	28.9	17.8	15	38.3
Sox9 A76E	28.3	14.8	15	42
Sox9 HMG	61.5	1	15.3	22.2
Sox9 WT + DNA	32.1	13.3	15.2	39.3
Sox9 A76E + DNA	31	16.8	16.9	35.4

Figure S3: Folding and secondary structure contents of Sox9 WT, Sox9 A76E and Sox9 HMG. (a) CD spectra of Sox9 WT and Sox9 A76E alone and in the presence of DNA oligo. (b) Table showing the percentage of secondary structures calculated from the CD spectra.

Figure S4.

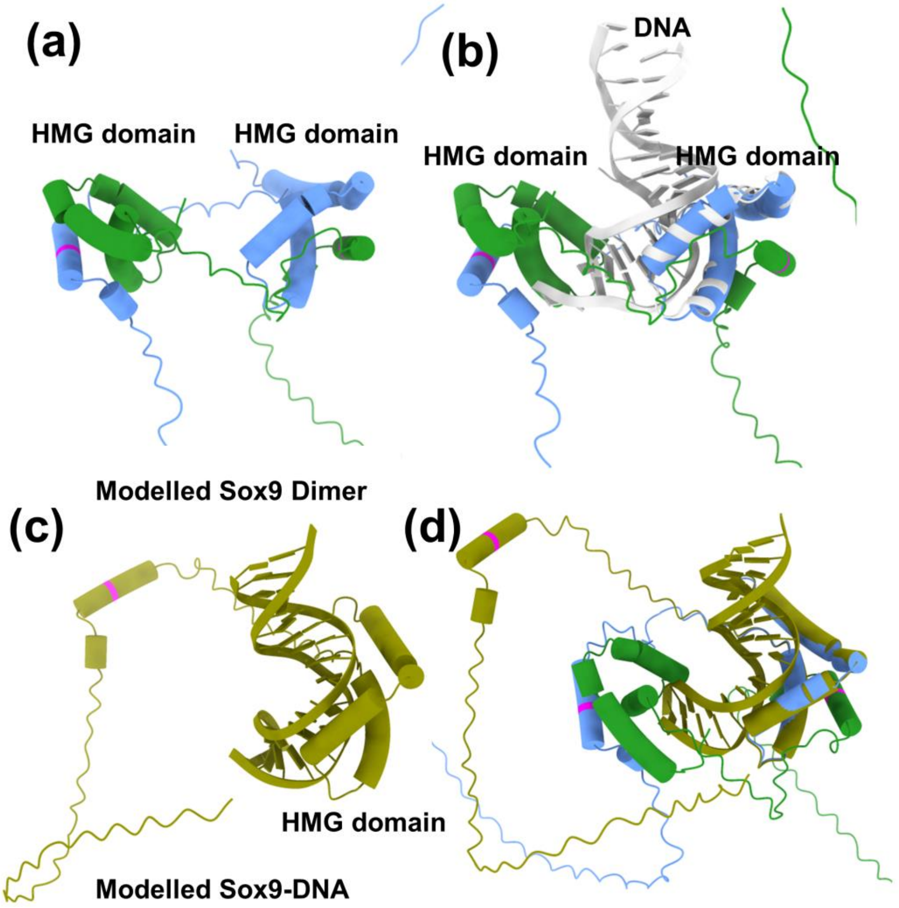

Figure S4: Structure models of Sox9 dimer and Sox9-DNA complex. (a) Cartoon representation of Sox9 dimer. Each monomer is shown in different colors (light blue and light green). (b) Structural superimposition of modelled Sox9 dimer with Sox9 HMG-DNA complex structure (PDB ID: 4S2Q). (c) Cartoon representation of modelled Sox9-DNA complex. (d) Structural superimposition of modelled Sox9 dimer with modelled Sox9-DNA complex structure.
